# Graduated Wine Glasses

**Published:** 1845-10-01

**Authors:** 


*Graduated Wine Glasses.*—L. S. Reynolds, Druggist, of this city, has received an article, new to us, entitled as above, which we think the profession would do well to recommend for families to provide themselves with. It is a wine glass of large size, graduated to administer tea-spoonful and table-spoonful doses of liquid medicines in the exact quantity intended to be expressed by these conventional terms of measure. Owing to variations of capacity in the table and tea spoons in common use, the quantities which these terms denote are very inexact, which, in several points of view, is an evil of not a little consequence. The introduction of these graduated glasses will establish an uniformity and precision in the administration of medicines which are very desirable.

